# High‐Performance Recycling Biobased Photopolymers for 3D Printing

**DOI:** 10.1002/advs.75006

**Published:** 2026-03-25

**Authors:** Hang Zhou, Yi Tan, Chuanwei Lu, Yanlin Li, Tianyi Tang, Zheng Yang, Ying Li, Xuehan Chen, Jianfeng Yao, Zhengchun Cai, Yuanyuan Jiang, Ping Zhao, Chengguo Liu

**Affiliations:** ^1^ College of Chemical Engineering Jiangsu Co‐Innovation Centre of Efficient Processing and Utilization of Forest Resources Jiangsu Key Lab for the Chemistry and Utilization of Agricultural and Forest Biomass Nanjing Forestry University Nanjing P. R. China; ^2^ Department of Orthodontics Nanjing Stomatological Hospital, Affiliated Hospital of Medical School Institute of Stomatology Nanjing University Nanjing P. R. China; ^3^ Key Laboratory of State Forestry and Grassland Administration on Highly‐Efficient Utilization of Forestry Biomass Resources in Southwest China Southwest Forestry University Kunming P. R. China

**Keywords:** 3D printing, biobased phenols, phenol‐carbamate bonds, photopolymers, recyclability

## Abstract

Developing recycling photo‐curing 3D printing materials (especially using renewable biobased feedstocks) can greatly alleviate environmental burden raised by the rapid development of 3D printing industry. Currently, it is still hard to achieve both high performance and excellent recyclability for such materials. In this study, by using a typical biobased phenol (i.e., eugenol) as starting material, we design biobased photopolymers containing dynamic dissociative phenol‐carbamate bonds, part of which come from the phenol itself. The obtained materials not only have relatively high biobased contents, but also demonstrate excellent thermal and mechanical properties. Subsequently, the materials are conveniently recycled by a proposed “mixed‐monomer assisted recycling” strategy, and high recovery efficiencies in at least three cycles were achieved. Furthermore, the materials indicate exceptional 3D printing performance and functionalities (such as oral bacteria inhibition and shape memory) as well as low environmental impacts. Overall, the combination of rigid biobased feedstocks (especially biobased phenols) and suitable dissociative covalent bonds offers the opportunity to develop high performance, recycling photopolymers with more functionalities and better sustainability.

## Graphical abstract

By using biobased phenols like eugenol, novel biobased photopolymers containing dissociative phenol‐carbamate bonds are designed for 3D printing. Meanwhile, a “mixed‐monomer assisted recycling” strategy is proposed to recycle the materials. The resulting materials not only achieve high‐performance and excellent chemical recyclability simultaneously, but also demonstrate high printing resolutions, superior anti‐bacteria effect, good shape memory and plasticity, and low environment impacts.

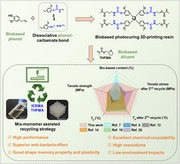

## 1 Introduction

Photo‐curing 3D printing technologies, such as stereolithography and digital lighting process (DLP) have been widely applied in industrial and biomedical areas due to their high resolution, free design, material saving, etc. [1, 2, 3]. The photocurable resins accompanied with these technologies are generally composed of photoactive prepolymer (i.e., crosslinker), diluting monomer, photoinitiator, and a few additives. After printing, the liquid resins form insoluble, infusible thermosetting polymers, which can be hardly recycled and reprocessed. In an effort to address this dilemma, dynamic covalent bonds (DCBs) have been introduced into photo‐curable materials. Initially, physical recycling of 3D printed items via grinding and hot pressing was reported [4, 5]. Nevertheless, this method turns valuable 3D printing materials into low‐value‐added sheets, which belongs to downcycling, thus researchers have been directed to chemical recycling of such materials.

The chemical recycling photopolymers can be classified into close‐loop and open‐loop materials according to whether the necessity of adding extra reactive species [6]. Machado et al. reported the first close‐loop recycling photopolymer enabling by dynamic cyclic disulfide, exhibiting a recovered resin yield up to 98% [7]. However, the inferior thermal/mechanical properties and slow polymerization rates of the prepared resins may restrict their real applications. In this year, Xie and coauthors developed another close‐loop recycling material using dissociative dithioacetal bond [8]. The optimal 3D‐printing sample achieved high recovered resin yield and superior toughness, while other mechanical and thermal properties were still moderate, e.g. maximum tensile strength and glass transition temperature (*T*_g_) of about 20 MPa and 25 °C, respectively. Currently, it is still very hard to achieve high‐performance close‐loop recycling resins, possibly because the cross‐linking sites formed via DCB reactions are limited or the explored DCBs are flexible. In comparison with the close‐loop materials, the open‐loop recycling materials can form numerous cross‐linking sites through polymerization of vinyl groups, and the DCBs can be selected without limit, thus it is more possible for them to overcome the obstacle.

According to the reaction mechanism, DCBs can be divided into associative and dissociative types: the later breaks during the reaction process, while the former don't [9]. In the past few years, several associative DCBs involving thioester bond [10], *β*‐hydroxyl ester bond [11], *β*‐carbonyl ester bond [12], and disulfide bond [13], have been employed to prepare the open‐loop recycling photopolymers. Nonetheless, the recycling process generally required high temperature and a long time, and the properties after recycling were significantly reduced. The reason lies in that the polymers based on associative DCBs are difficult to be decomposed and achieve fast solid–liquid conversion. On the contrary, polymers bearing dissociative DCBs have inherent advantages in network decomposition and solid–liquid conversion. On account of these merits, we reported a recycling photopolymer based on hindered urea bond (HUB), a typical dissociative DCB, and recycled the material conveniently via a “monomer‐assisted recycling” strategy (only taking 2 h for complete degradation at 100°C) [14]. Notably, the physicochemical properties, including viscosities, polymerization rates, and thermal/mechanical properties of the resins recycled three times were almost identical to those of the original one. Nevertheless, developing such materials with both high performance and excellent recyclability also remains a huge challenge.

**FIGURE 1:**
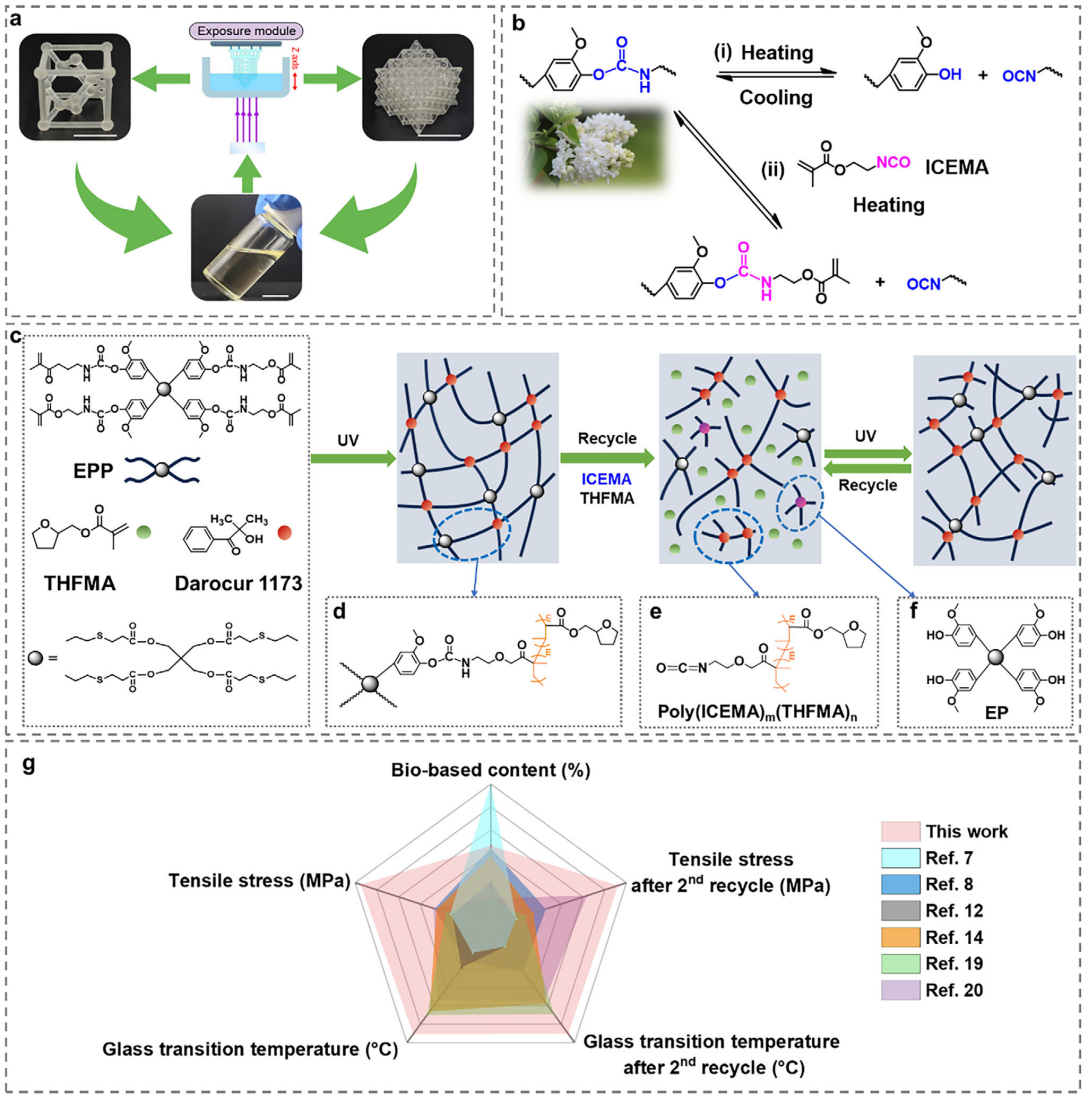
Recycling 3D printing biobased photopolymers based on phenol‐carbamate bonds (PCBs). (a) Graphs of biobased photocurable resins in DLP printing, chemical dissolution, and reprinting (Scale bar: 1 cm). (b) Eugenol‐based dynamic PCB reactions under dissociative mechanism (i) without extra reactants and (ii) with extra ICEMA monomer. (c) Illustration of the structural evolution of biobased PCB‐containing resins after UV curing (or printing), recycling, and UV curing again (or reprinting). (d–f) Chemical structures of the resultant crosslinked networks after printing and recycling. (g) Comparison of the performance of this work with other recycling photopolymers [7, 8, 12, 14, 19, 20].

On the other hand, due to the crises involving the depletion of petroleum resources, the greenhouse effect, and environmental pollution, the employment of renewable biobased resources has become a top priority for the development of photopolymers [15]. Biobased phenols are a kind of important biobased resources, which have various origins like lignin (the second largest biomass resource after cellulose), cashew nut, raw lacquer, etc [16]. Lignin‐derived phenols such as eugenol and vanillin have become preferred candidates for the preparation of high‐performance polymers ascribed to the inherent rigid benzene rings [17]. Specially, the intrinsic phenolic hydroxyls can also provide an ideal platform for the construction of dynamic phenol‐carbamate bond (PCB), of which the dissociation reaction occurs under mild condition [18]. Therefore, in this study, we designed eugenol‐based photopolymers containing partially intrinsic PCBs for 3D printing (Figure 1). It should be mentioned that the dissociative PCB was employed in the design of a recyclable photopolymer for the first time. The obtained materials demonstrated superior mechanical and thermal properties, with maximum tensile strength and *T*_g_ of 68.3 MPa and 83.2°C, respectively, comparable or superior to a commercial counterpart. Meanwhile, the optimal material could be conveniently recycled via a “mixed‐monomer assisted recycling” strategy (Figure 1c–f), and the recycled resins achieved remarkable recycling efficiencies of thermal and mechanical properties simultaneously. As indicated in the comparative radar chart (Figure 1g), the optimal product outperformed analogous recycling photopolymers in terms of mechanical strength and *T*_g_ [7, 8, 12, 14, 19, 20]. Furthermore, 3D printing performance and functionalities involving antibacterial, shape memory, and plasticizing properties as well as life‐cycle assessment of the biobased materials, were also investigated.

## 2 Results

### 2.1 Synthesis and Properties of Eugenol‐Based Photocurable Resins

The synthesis of eugenol‐based PCB‐containing prepolymers involves two steps (Figure S1a,b): Firstly, eugenol reacted with sulfhydryl compounds such as 2,2′‐(ethylenedioxy) diethanethiol and pentaerythritol tetra(3‐mercaptopropionate) (EDDET and PETMP) via “thiol–ene” photo‐click reaction to produce eugenol‐based phenols (ED and EP). Secondly, the obtained phenols were reacted with 2‐isocyanatoethyl methacrylate (ICEMA) in the presence of dibutyltin dilaurate as a catalyst to produce two prepolymers (EDP and EPP) with a theoretical carbon–carbon double bond (C = C) functionality of 2 and 4. Experimental conditions, including reaction time and feed ratio were investigated to obtain the optimal condition. As shown in Figure S2, FT‐IR technique was employed to monitor the conversion of isocyanate (‐NCO) group at around 2250 cm^−1^ during the reaction of EP and ICEMA with a molar ratio of EP:ICEMA = 1:4. The ‐NCO peak completely disappeared after 4 h, indicating full conversion of ‐NCO group. Subsequently, mechanical properties of the resins with different feed ratio of EP and ICEMA were examined (Figure S3). As the molar ratio of EP to ICEMA changed from 1:4 to 1:4.3, the tensile strength of the resultant material decreased from 72.1 to 37.3 MPa. The reason probably lies in that ICEMA can completely react with phenolic hydroxyl groups on EP to form a uniform crosslinked network at the ratio of 1:4, while excessive ICEMA leads to a plasticizing effect or phase separation in the matrix [21]. Therefore, the reaction time of 4 h and feed ratio of EP:ICEMA = 1:4 were selected as the optimal conditions for the synthesis of prepolymers. As for structural characterization, FT‐IR and ^1^H NMR spectra of eugenol, eugenol‐based phenols, and eugenol‐based prepolymers are provided in Figures S4–S10.

**FIGURE 2:**
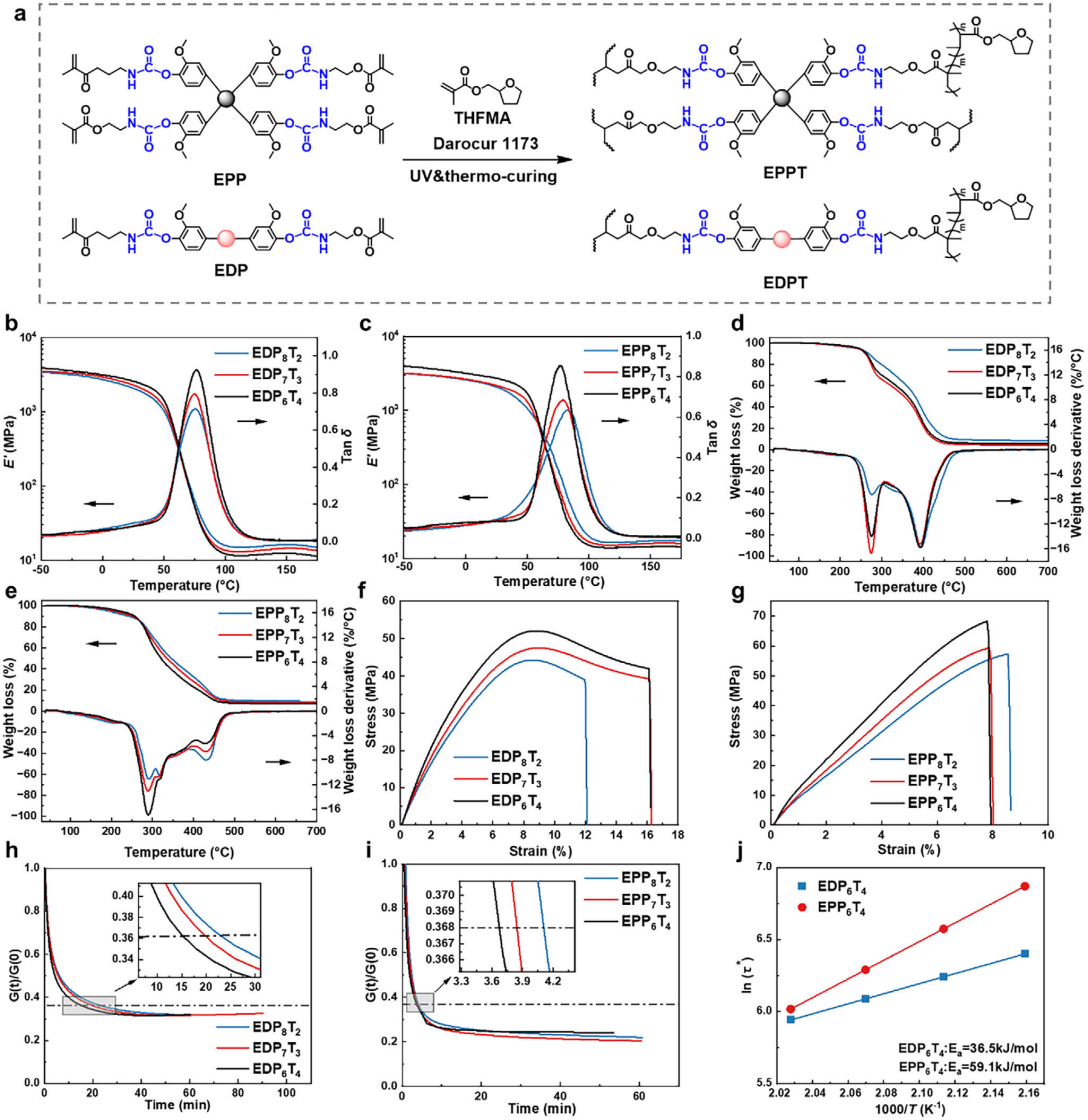
Comprehensive thermo‐mechanical and stress–relaxation properties of EDPT and EPPT. (a) Preparation and curing of biobased PCB‐containing resins. (b, c) Storage modulus and loss factor, (d, e) TGA and DTG curves, (f, g) Typical stress–strain curves, and (h, i) stress relaxation curves of EDPT and EPPT; (j) ln (τ*) vs. 1000/*T* plot of EDP_6_T_4_ and EPP_6_T_4_.

The biobased UV‐curable resins were prepared by blending the obtained EDP/EPP with tetrahydrofurfuryl methacrylate (THFMA), a biobased diluent, and Darocur 1173 photoinitiator (Figure 2a and Table S1). Physical properties, including viscosity (*V*_s_), gel content (*C*_gel_), biobased content (*C*_bio_), and volumetric shrinkage (Δ*V*) were tested (Table S2). Firstly, the *V*_s_ is a critical parameter for evaluating the applicability of UV‐curable resins in 3D printing. Commercial UV‐curing 3D printing systems typically require the *V*_s_ of a resin lower than 1300 mPa s to ensure recoating a layer smoothly and curing accurately [22]. The *V*_s_ of EDPT and EPPT ranged from 103 to 580 mPa s, indicating excellent suitability for UV‐curing 3D printing. The *V*_s_ gradually decreased as the content of THFMA increased, showing the feasibility of using THFMA as a reactive diluent for tailoring the fluidity of resin. Secondly, all the resins showed a *C*_gel_ value exceeding 95%, indicating a high crosslinking extent of the polymer networks. The EPPT samples demonstrated higher *C*_gel_ values than the EDPT samples, which is probably due to that the functionality of EPP is higher than that of EDP. The higher THFMA content also led to the growth of *C*_gel_, which may be caused by the enhanced diffusion and better network formation under reduced *V*_s_. As for the *C*_bio_ (defined as mass percentage of biobased carbon to total organic carbon in a product), all the values were above 40%, demonstrating relatively high biobased content. Finally, the Δ*V* values for EDPT resins remained almost unchanged, with a value of around 9.0%, while these values for EPPT dropped from 15.9% at 20% of THFMA to 8.83% at 40% of THFMA, which is possibly attributed to the drop of C = C content in the systems [12].

UV‐curing kinetics of the EDPT and EPPT formulations were further investigated via the real‐time infrared (RT‐IR) technique. The time‐dependent evolution of C = C conversions and their corresponding rates are presented in Figure S11, and the kinetic parameters, including final C = C conversion (*α*_f_) and maximum C = C conversion rate (*R*_p_) are summarized in Table S2. The *R*_p_ values of the resins occurred within 15 s under irradiation, indicating a rapid photopolymerization process favorable for UV‐curing 3D printing [23]. As the growth of THF content, the *α*_f_ increased from 63.5% to 82.3% in EDPT and from 65.3% to 75.7% in EPPT, and *R*_p_ rose from 0.114 to 0.182 s^−1^ in EDPT and from 0.0780 to 0.130 s^−1^ in EPPT. The enhancement in *α*_f_ may stem from improved chain mobility and segmental diffusion, as increasing THFMA content reduces *V*_s_ and facilitates the motion of the terminal C = C groups in the prepolymer chains. However, the increase of *R*_p_ can be attributed to that the addition of more THFMA raises the concentration of reactive C = C groups in the systems [24].

Dynamic mechanical analysis (DMA) was employed to detect thermo‐mechanical properties of the cured materials. Curves of storage modulus and loss factor (tan* δ*) vs. temperature are plotted in in Figure 2b,c, and the corresponding data are summarized in Table S3. Glass transition temperature (*T*_g_) is determined from the peak of tan* δ* curve, and cross‐link density (*ν*_e_) is calculated based on rubber elasticity theory using the following equation:[25]

(1) $$\nu_{e}=\frac{E^{\prime }}{3RT}\;$$

where *E*’ is the storage modulus in the rubbery plateau region (at *T*_g_ + 40°C), *R* is the universal gas constant, and *T* is the absolute temperature. First, the samples demonstrated a storage modulus at 25°C (*E’*_25_) and *T*_g_ in the range of 2.05–2.50 GPa and 73.6–83.2°C, which were very high among the reported similar bio‐based systems [26, 27, 28, 29]. Second, both of the storage modulus at 25°C (*E’*_25_) and *T*_g_ values increased basically with the growth of THFMA content. Generally, *E’*_25_ and *T*_g_ of thermosets increase as the growth of *ν*_e_ or hard segment content (*C*_HS_), and vice versa [25, 30, 31, 32]. When THFMA content increased, *ν*_e_ decreased while *C*_HS_ increased (Table S3), suggesting the growing trend of these properties is mainly attributed to the *C*_HS_. Besides, the EPPT resins demonstrated higher *E’*_25_ and *T*_g_ than the EDPT ones, which can be ascribed to the higher C = C functionality of the EPP prepolymer. Thermogravimetric analysis (TGA) was further utilized to analyze the thermal stability of the obtained materials. Weight–loss curves and their derivatives are presented in Figure 2d,e, and the related data are also listed in Table S3. Firstly, 5% weight loss temperature (*T*_5_), denoting the onset decomposition temperature, corresponded to the volatilization of unreacted species (e.g., residual catalyst) and partial cleavage of liable urethane bonds in polyurethane systems [33]. As the growth of THFMA content, the *T*_5_ values increased gradually for both EDPT and EPPT, which can be ascribed to the decreasing content of dynamic PCBs (Table S3). The EDPT resins showed higher *T*_5_ values than the EPPT ones, which is probably due to the less contents of PCBs in the EDPT materials. Secondly, the first and second maximum decomposition temperatures (*T*_max1_ and *T*_max2_) were mainly correspond to the decomposition and charformation of the urethane linkages/ester groups and soft segments like thiol components, respectively. Both the *T*_max1_ and *T*_max2_ remained almost unchanged for EDPT and EPPT when the THFMA content increased. However, the *T*_max1_ and *T*_max2_ values of EPPT were clearly larger than those of EDPT, which is possibly because EPPT possesses higher ester group content and soft segment content (indicated by *C*_HS_). It is worth mentioning that all the materials exhibited high *T*_5_, *T*_max1_, and *T*_max2_ values compared with the similar biobased UV‐curable systems [26, 27, 28, 29], reflecting good thermal resistance. As for mechanical properties, typical stress–strain behaviors of the EDPT and EPPT resins are depicted in Figure 2f,g, and the related results are summarized in Table S4. Firstly, when the THFMA content increased from 20% to 40%, the tensile strength (*σ*) and Young's modulus (*E*) of the EPPT material grew from 54.7 to 68.0 MPa and from 0.941 to 1.41 GPa, respectively. A similar trend was demonstrated for the EDPT materials. The reason for these trends lies in that the *C*_HS_ values increase in the two systems [25, 30]. Besides, the EPPT systems showed apparently larger *σ* and *E* values than the EDPT systems, which is mainly attributed to the high *ν*_e_ of EPPT. Notably, the EPP_6_T_4_ sample achieved the *σ* and *T_g_* of 68.0 MPa and 83.2 °C, respectively, which were comparable or superior to a commercial counterpart (Figures S12 and S13 and Table S5).

Stress relaxation experiments confirmed the presence of thermally activated DCBs in the cured resins, as shown in Figure 2h,i. According to the Maxwell model, the relaxation time (τ*) is defined as the time at which the stress decreased to 1/e of the initial value. The τ* values decreased by increasing THFMA content (Tables S6 and S7), from 21.9 to 15.5 min at 180 °C for EDPT, and from 4.31 to 3.71 min at 200 °C for EPPT. The relaxation time is usually affected by the structural factors, including *ν*_e_, *C*_HS_, and DCB content (*C*_DCB_): The rise of *C*_DCB_ or the drop of *ν*_e_/*C*_HS_ usually leads to the decrease of τ*, and vice versa [17, 34, 35, 36]. As the growth of THF content, both the *C*_DCB_ and *ν*_e_ dropped while the *C*_HS_ rose (Tables S2 and S3), thus the decrease of τ* can be mainly attributed to the drop of *ν*_e_ in EDPT and EPPT. Activation energy (*E*_a_) is another important parameter to characterize DCB reactions in the polymer networks. The activation energy (*E*_a_) values were calculated according to the Arrhenius equation:[17, 34, 35, 36]

(2) $$\ln\;\tau^{*}=\frac{E_{a}}{\mathrm{RT}}-\ln A$$

where *A* is the pre‐exponential factor, and *R*, *T* are identical to those shown in Equation (1). EDP_6_T_4_ and EPP_6_T_4_ were selected to determine the *E*_a_ values as they show the lowest τ* values (Figure  2j and Figure S14). The calculated *E*_a_ values were 36.5 kJ mol^−1^ for EDP_6_T_4_ and 59.1 kJ mol^−1^ for EPP_6_T_4_, respectively. The low *E*_a_ values indicated reduced energy barrier for bond exchange and faster reaction rates, favorable for superior self‐repairability, recyclability, etc [37]. In addition, in order to study the durability of the obtained materials, creep tests of EPP_6_T_4_ were conducted at different temperature with a stress of 0.5 MPa. As shown in Figures S15 and S16, EPP_6_T_4_ exhibited negligible creep (residual strain ≈ 0) at 40°C and a little creep (residual strain < 0.8%) at 50–60°C. However, when the temperature increased to higher temperatures (80–100°C), the residual strains reached 2.5%–3.1%, suggesting the creep resistance at high temperatures needs to be improved.

### 2.2 Recycling Mechanism and Performance

The EPP_6_T_4_ sample was selected as the optimal sample to perform the following studies since it showed the best mechanical and thermal properties (represented by *σ* and *T*_g_) among all the samples and the lowest τ* value in the EPPT materials. Conventional recycling method of UV‐curing 3D printing material usually involves utilizing a compound without a photoactive group (e.g., ethylene glycol) to degrade the printed item first and then react the degraded resin with a photoactive compound to form printable resin [11, 12, 38]. In order to simplify this method, we had invented the “monomer‐assisted recycling” strategy to recycle a HUB‐containing UV‐curing 3D printing material, as mentioned above [39]. The printed item was degraded directly under 2‐(*tert* butylamino) ethyl methacrylate (TBEM) monomer to form a new reprintable resin. However, the TBEM is petroleum‐based, expensive, and not commonly used as diluent, thus a mixture of ICEMA and biobased THFMA monomers was used to recycle the cured materials in this work (Figure 1 and Movie S1), which is named the “mixed‐monomer assisted recycling” strategy. In this strategy, ICEMA is used for the degradation of photopolymer, while THFMA only acts as a solvent (in the later curing stage, it works as a diluent). Notably, the EPP_6_T_4_ could be totally degraded in the ICEMA/THFMA mixture at 110°C for only 2 h, indicating a fast recycling rate and mild recycling conditions [40, 41]. The recovery efficiency of material mass, defined as mass percentage of the original material in the recycled resin, was 27.5% for the 1st and 2nd cycles and 22.9% for the 3rd cycle, respectively. Compared with other open‐loop recycling systems [11, 14, 19], the recycling efficiencies of material mass achieved in this work were clearly higher (Table S8).

**FIGURE 3:**
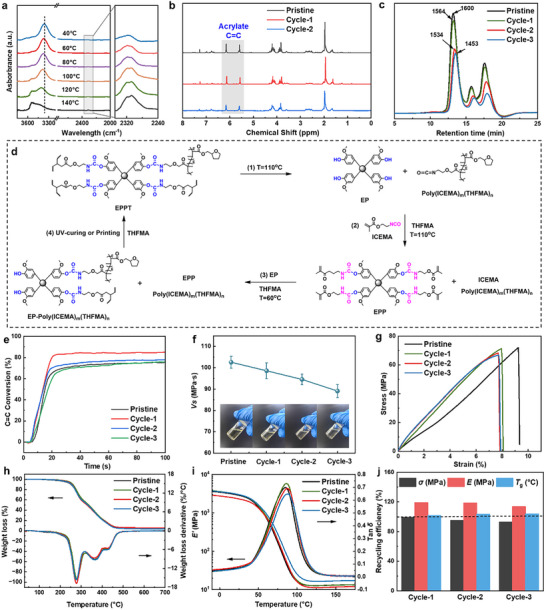
Chemical recycling and property retention of EPP_6_T_4_. (a) Temperature‐dependent FT‐IR spectra of EPP_6_T_4_ from 40 to 140°C. (b) ^1^H NMR spectra and (c) GPC curves of the pristine and recycled resins. (d) Proposed reaction mechanism of EPP_6_T_4_ during the recycling process. (e) C = C conversions, (f) viscosities, (g) stress–strain curves, (h) TGA and DTG curves, and (i) storage modulus and loss factor curves of the pristine and recycled resins. (j) Recycling efficiencies of tensile strength (*σ*), Young's modulus (*E*), and *T*_g_.

Temperature‐dependent IR spectra of EPP_6_T_4_ within a range of 40–140°C (Figure 3a) captured the ‐OH band shifting from 3362 to 3549 cm^−1^ and broadening concurrent with the appearance of –NCO absorption at around 2306 cm^−1^ demonstrating covalent bond scission and hydrogen‐bond disruption [40]. Gel permeation chromatography (GPC) and ^1^H NMR were used to analyze the structures of the recycled EPP_6_T_4_ resins, as shown in Figure 3b,c, and related data are listed in Table S9. All the GPC curves of the recycled resins overlapped closely with that of the pristine one, and all the data of molecular weights (*M*_w_, *M*_n_, and polydispersity) varied slightly with increasing cycle times, confirming the excellent structural stability and reprocessability of EPP_6_T_4_ during recycling. In the ^1^H NMR spectra, the peaks at around 5.6 and 6.2 ppm are attributed to acrylate C = C protons, indicating the presence of photoactive groups similar to those in the original resin. The remaining characteristic peaks also closely matched those of the pristine resin, suggesting that the recycled EPP_6_T_4_ preserves the essential chemical structures of the original resin. Based on the above results, we can deduce that the EPPT material probably experienced a three‐step stages during the recycling process (Figure 3d): First, the EPPT material underwent reversible dissociation under heating and produced degraded products such as EP and poly(ICEMA)_m_(THFMA)_n_; Second, the yielding EP reacted with ICEMA to form EPP prepolymer, while poly(ICEMA)_m_(THFMA)_n_ remained un‐changed; Finally, a certain amount of fresh EP was supplemented and partially reacted with the residual isocyanate groups in the system, which can be totally reacted during the curing stage.

Photopolymerization kinetics of the recycled resins were further investigated (Figure 3e), with detailed data listed in Table S9. The *α*_f_ values of the recycled resins were in a range of 77.5%–83.8%, all higher than that of the pristine resin, which is due to the addition of excessive THFMA. The *R*_p_ values of the recycled resins were lower than that of the pristine resin, however, the highest value reached 0.128 s^−1^ in the 2nd recycle, very close to the data of the pristine resin. These results demonstrated that EPP_6_T_4_ maintained efficient photoreactivity after multiple recycling cycles, further confirming its excellent recyclability and structural integrity. The viscosity of recycled resin (Figure 3f) decreased with the recycling number, suggesting the restoration of a better 3D‐printable liquid resin after regeneration, and the recovered resins also showed a clear, transparent liquid state similar to the original one. Tensile stress–strain curves (Figure 3g) further demonstrated excellent retention of mechanical performance, as the tensile strengths after the 1st to 3rd cycles were 71.7, 68.7, and 67.4 MPa, approaching the original value (72.5 MPa), with recovery efficiencies up to 99.7%, 94.9%, and 93.1%, respectively (Table S9). The Young's modulus recovery efficiencies were 118.7%, 118.7%, and 114.1%, respectively. Thermal stability assessed by TGA (Figure 3h and Table S10) further confirmed the thermal robustness of the recycled materials. The *T*_5_, *T*_max1_, and *T*_max2_ nearly unchanged after recycling, suggesting retained thermal stability. DMA results (Figure 3i and Table S10) revealed that the *T_g_* of the recycled resins (1st, 2nd, and 3rd cycles) were 86.7, 87.5, and 88.7°C, slightly higher than that of the pristine (83.2°C), with recovery efficiencies of 101.7%, 103.6%, and 104.3%, respectively. Accompanied by a progressive enhancement in *E'*_25_ from 2.40 to 2.81 GPa across recycling cycles, the *ν_e_* exhibited a gradual increase from 1.40 × 10^3^ to 1.56 × 10^3^ mol m^−3^ which contributes to the elevated mechanical and thermal performance even after multiple recycles. A comparative bar‐chart summary of recovery metrics is provided in Figure 3j, indicating that all the recovery efficiencies of mechanical and thermal properties fluctuated at 100%.

### 2.3 3D‐Printing Performance, Functionality, and Life‐Cycle Assessment

**FIGURE 4:**
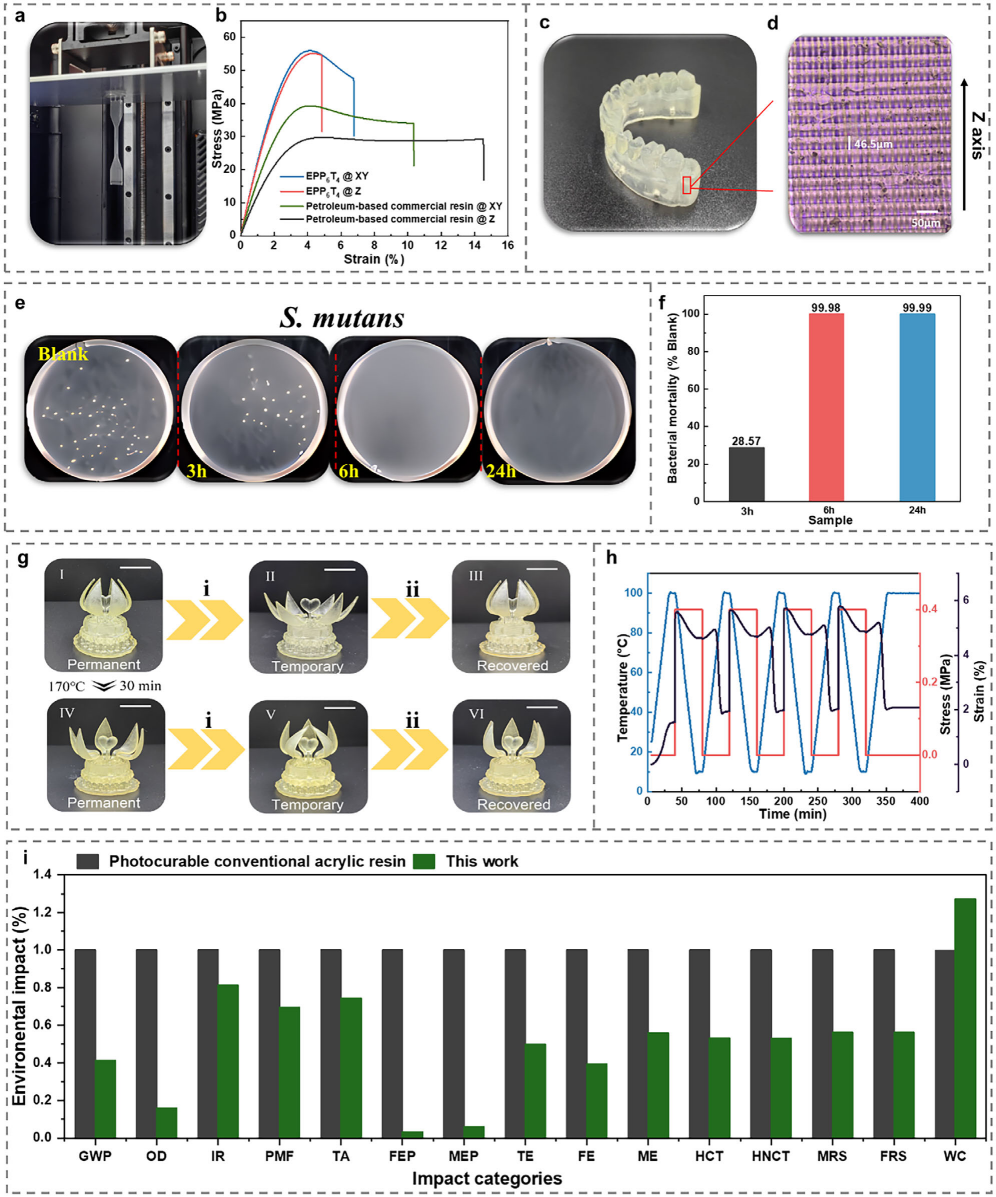
3D printing performance, multi‐functionality, and environmental assessment of EPP_6_T_4_. (a) Photograph of 3D printed specimen for mechanical testing. (b) Stress–strain curves of EPP_6_T_4_ and commercial resin 3D printed samples in both XY‐ and Z‐directions. (c) Photograph of 3D printed brace using EPP_6_T_4_. (d) Z‐axis accuracy of 3D printed braces using EPP_6_T_4_. (e) Antibacterial effect of EPP_6_T_4_ against *Streptococcus mutans* in 3, 6, and 24 h, with the blank group as control. (f) Antibacterial rate of EPP_6_T_4_. (g) Visual demonstration of temporary and permanent shape change performance, where (i) represents the shape change process at 90°C and the shape fixation process in cold water, and (ii) represents the shape recovery process at 90°C (Scale bar: 4 cm). (h) Consecutive dual‐shape memory cycles of the EPP_6_T_4_. (i) Normalized comparison of environmental impacts between EPP_6_T_4_ and a photocurable conventional acrylic resin [46]. Abbreviations: global warming potential (GWP), ozone depletion (OD), ionizing radiation (IR), particle matter formation (PMF), terrestrial acidification (TA), freshwater eutrophication potential (FEP), marine eutrophication potential (MEP), terrestrial ecotoxicity (TE), freshwater ecotoxicity (FE), marine ecotoxicity (ME), human carcinogenic toxicity (HCT), human noncarcinogenic toxicity (HNCT), mineral resource scarcity (MRS), fossil resource scarcity (FRS), and water consumption (WC).

The practical applicability of the EPP_6_T_4_ was further demonstrated via 3D printing, as shown in Figure 4. Dog‐bone specimens printed along both the XY‐ and Z‐axes exhibited excellent mechanical performance, with tensile strengths of 56.1 and 55.3 MPa (Figure 4a,b and Table S11), respectively, significantly outperforming those of the commercial counterpart (39.3 and 29.7 MPa for the XY‐ and Z‐axes, respectively). It is worth mentioning that the EPP_6_T_4_ possessed superior isotropy and interlayer adhesion, which are critical for structural reliability in dental applications. To further demonstrate the accuracies, a customized dental aligner was fabricated using EPP_6_T_4_ (Figure 4c), of which the actual Z‐layer thickness (46.5 µm) was close to the designed specification (50 µm, Figure 4d), while the measured minimum printing precision of the XY‐axis could reach 53.1 µm (Figure S17), indicating that the spatial resolutions in all directions were very high. Such dimensional fidelity is essential for orthodontic applications, where clinical outcomes rely heavily on microscale accuracy [42, 43]. Besides, antibacterial activity of the cured EPP_6_T_4_ was evaluated. The *Streptococcus mutans* was chosen as test organism because it accounts for the largest proportion of natural oral microbiota and is one of the main components of dental plaques. As shown in Figure 4e,f, the surface bacterial colonies on the EPP_6_T_4_ reduced by 28.6% after 3 h compared to the blank control, and this inhibition exceeded 99.9% after 6 h, manifesting the potent bacteriostatic efficacy derived from the retained phenolic ‐OH in EPP_6_T_4_ [44]. *Streptococcus mutans* is particularly susceptible to phenolic compounds, which can disrupt cell membrane integrity and inhibit key metabolic enzymes [45]. In addition, cytotoxicity tests were also performed by separately measuring the numbers of live and dead cells, as depicted in Figures S18 and S19. The two tests, consistent with each other, showed no significant difference compared with the control group, which confirmed the favorable biocompatibility of EPP_6_T_4_. Incorporation of eugenol into the crosslinked network preserves the sustained antibacterial action without compromising the mechanical stability, demonstrating the potential of EPP_6_T_4_ for antimicrobial dental and other biomedical applications.

UV‐induced crosslinked networks typically impart favorable mechanical performance to cured materials, while dynamic covalent exchange reactions at elevated temperatures confer plasticity and multiple shape memory functionalities. To demonstrate these functionalities of the EPP_6_T_4_ material, a lotus flower shape is designed and fabricated via 3D printing. As shown in Figure 4g and Movie S2, the flower bud shape (I) was temporarily deformed into shape (II) at a temperature slightly above *T*_g_ (90°C) and then fixed by immersion in cold water. Upon reheating at 90°C in process (ii), it could recover to the original flower bud shape (III) within approximately 5 min, exhibiting excellent shape recovery performance, which is primarily governed by the permanent crosslinked network. Separately, the original flower bud sample could be permanently deformed into a spiral shape (IV) at 170°C for 30 min, benefiting from the superior plasticity from PCBs. Subsequently, it could be temporarily reshaped into sample (V) at 90°C and fixed in cold water. When reheated at 90°C, the shape (V) could gradually return to shape (VI) like shape (IV), rather than the original flower bud shape (I), indicating good shape memory even after plastic deformation. To further implement quantization of the shape memory, the continuous shape memory curves of EPP_6_T_4_ are presented in Figure 4h, with corresponding data listed in Table S12. The shape fixity ratio (*R*_f_) and recovery ratio (*R*_r_) were calculated by the following equations:

(3) $$R_{f}=\frac{\varepsilon_{\mathrm{unload}}}{\varepsilon_{\mathrm{load}}}\;\times 100\%$$

(4) $$R_{r}=\frac{\varepsilon_{\mathrm{unload}}-\varepsilon_{\mathrm{rec}}}{\varepsilon_{\mathrm{unload}}}\;\times 100\%$$

where *ε*_unload_, *ε*_load_, and *ε*_rec_ represent the fixed strain, initial strain, and recovery strain of each cycle, respectively. The *R*_f_ values were all above 80%, benefiting from the limited relative polymer chain mobility due to the substantial difference between the glassy and rubbery moduli [47, 48]. Besides, the *R*_r_ values within the four cycles were in a range of 59.5%–65.0%, which is due to the dynamic rearrangements of PCBs at elevated temperatures that can partially alter the network structure [47, 48]. The results confirmed that the EPP_6_T_4_ exhibited a robust combination of permanent network‐driven shape fixation and dynamic bond‐enabled adaptability, rendering it a versatile candidate for applications requiring both precise shape memory and reprogrammable plasticity.

A life‐cycle assessment (LCA) was performed to assess the environmental impacts of the proposed process, under the assumption that the resin undergoes three recycling cycles. The system boundary is depicted in Figure S20, and detailed LCA data are provided in Tables S13–S15. A functional unit of 1 kg of EPP_6_T_4_ resin was adopted, and its life‐cycle burdens were compared with those of a photocurable conventional acrylic resin [46]. The results (Figure 4i) indicated that our process possessed environmental benefits almost across all fifteen assessed categories except water consumption.

## 3 Conclusions

In this work, novel high‐performance, recycling photopolymers for 3D printing were successfully fabricated through the integration of dynamic dissociative PCBs and rigid biobased feedstocks like phenols and furans. The high performance can be attributed to both the high hard segment content and cross‐link density of the obtained materials. Meanwhile, the rapid recycling rates under relatively mild conditions and exceptional recycling efficiencies indicated that the developed “mixed‐monomer assisted recycling” strategy is effective and sustainable for open‐loop recycling of photopolymers bearing dissociative DCBs. In addition, the materials exhibited superior 3D printing accuracies, muti‐functionalities such as anti‐bacteria and shape memory, and low environment impacts. Despite these virtues, a key limitation of such materials is the inferior dimension stability at high temperatures (≥80 °C), which is possibly caused by the relatively low activation energy of PCB. Future work will explore dissociative DCB‐containing systems with higher activation energies. In general, this study paves the way of designing high‐performance, recycling, and functional photopolymers from rigid biobased feedstocks, which can greatly alleviate environmental challenges resulting from 3D printing materials and realize the valorization of numerous biobased resources.

## 4 Experimental Section

### 4.1 Materials

Eugenol (>98%), ethyl acetate (EA, ≥99.5%), and anhydrous magnesium sulfate were provided by Nanjing Reagent Co., Ltd. (China). The EDDET (>99%) and PETMP (>99%) were offered by Sigma–Aldrich Co., Ltd. (USA). The ICEMA (>99%), THFMA (>96%), ditin butyl dilaurate (DBTDL, >95%), 2‐hydroxy‐4′‐(2‐hydroxyethoxy)‐2‐methylpropiophenone (Irgacure 2959), and 2‐hydroxy‐2‐methyl‐1‐phenylpropan‐1‐one (Darocur 1173) were obtained from Aladdin Co., Ltd. (China). Before synthesis, eugenol, EDDET, PETMP, ICEMA, and THFMA were all dried by molecular sieves for one week. The commercial photo‐curing 3D printing resin was supplied by Anycubic Corporation (China).

### 4.2 Synthesis of Eugenol‐Based Prepolymers

The eugenol‐based photosensitive prepolymers were synthesized via “thiol–ene” photo‐click reaction and isocyanation reaction, as shown in Figure S1. Typically, eugenol (32.84 g, 0.2 mol), PETMP (24.43 g, 0.05 mol), and Irgacure 2959 photoinitiator (1.14 g) were firstly added to a 100 mL flask and stirred under N_2_ atmosphere and UV lighting (*λ*  = 365 nm) for 1 h to obtain the eugenol‐based tetraphenol (EP). Subsequently, EP (50.0 g, 0.0437 mol), ICEMA (27.01 g, 0.174 mol), and EA (100 mL) were charged into a 250 mL four‐neck flask and heated to 60°C under *N*_2_ atmosphere, then DBTDL (0.77 g, 1 wt.%) was added dropwise and reacted for 4 h. Upon completion, the crude product was washed three times with brine and distilled water, respectively. The organic layer was dried by anhydrous magnesium sulfate, filtered, and concentrated under reduced pressure to yield the polyurethane acylate prepolymer (EPP) with a theoretical C = C functionality of 4. The prepolymer (EDP) based on EDDET with a theoretical functionality of 2 was synthesized similarly.

### 4.3 Preparation of UV‐Curing 3D Printing Biobased Resins

UV‐curable 3D printing resins were prepared by mixing the synthesized EDP or EPP with THFMA (20%–40% of total weight of the prepolymer and diluent) and Darocur 1173 (2% of total weight of the prepolymer and diluent), as listed in Table S1. For simplicity, the obtained samples were labeled as EDP_x_T_y_ and EPP_x_T_y_, in which x and y denote the proportions of the prepolymer and diluent used in each sample. For instance, the EDP_x_T_y_ means the sample contains x parts of EDP and y parts of THFMA. The mixtures were stirred in a beaker for 5 min, degassed under vacuum for 30 min, and cast into polytetrafluoroethylene (PTFE) molds with various shapes. For the evaluation of mechanical, thermal, and stress relaxation properties as well as recycling performance, the cast resins were cured using an Intelli‐Ray 400 UV‐curing system (Uvitron International Corporation, USA) at a wavelength of 365 nm and an intensity of 100 W cm^−2^ for 60 s. Subsequently, the samples were thermally postcured at 140°C for 4 h. For DLP‐based 3D printing, models were pre‐processed using Photon Workshop software to add support structures and sliced according to the printer specifications. The EPP_6_T_4_ liquid resin was added to the vat of a Photon Ultra DLP printer (Anycubic Corporation, China). The setting thickness per layer was 50 µm and the exposure time per layer was 10 s. After printing, the objects were washed with ethanol to remove the uncured resin, followed by a post‐curing step (300 s) using an HTBX‐II UV curing chamber (Height‐LED Corporation, China) at an intensity of 100 W cm^−2^. The XY‐axis resolution test was performed on a microArch S150 3D printer (BMF Nano Material Technology, China) according to a similar procedure.

### 4.4 Recycling of Cured or Printed Biobased Resins

To implement the recyclability, a “mixed‐monomer assisted recycling” strategy was employed to recycle the EPP_6_T_4_. Typically, 4.05 g of the cured EPP_6_T_4_ (obtained from 2.38 g of EPP and 1.59 g of THFMA) was placed in a 25 mL three‐neck flask. Subsequently, ICEMA (1.69 g, 10.8 mmol) and THFMA (5.89 g) were introduced into the flask and stirred at 110°C under N_2_. The solid resin could be fully dissolved within 2 h. After cooling to 60°C, EP (3.10 g, 2.70 mmol) was added into the degraded resin and reacted for 2 h to neutralize the excess isocyanate groups, then a liquid printable resin was obtained. In the third cycle, the THFMA increased to 50 wt.% of the total recycled resin. The recycling procedures of printed items were similar to the above process (Movie S1).

### 4.5 Characterization

All the FT‐IR spectra were conducted using a Nicolet iS10 IR spectrometer from Thermo‐Fisher Cooperation (USA). The samples were scanned from 4000 to 500 cm^−1^ with a resolution of 4 cm^−1^. All the ^1^H NMR spectra were carried out on a DRX‐300 Advance NMR spectrometer from Bruker Corporation (Germany) by employing deuterated chloroform (CDCl_3_) as solvent. Real‐time Infrared (RT‐IR) spectroscopy was recorded on a modified Nicolet 5700 spectrometer from Thermo‐Nicolet Instrument Corporation (USA) with an exposure intensity of 100 W cm^−2^. The kinetic parameters were determined by monitoring the intensity of the peak at 810 cm^−1^. Soxhlet extraction equipment was employed to determine the gel contents according to the procedures listed in our previous works [25, 49]. Volumetric shrinkage was determined using an FZMD‐2 electronic densitometer (Shanghai Fangrui Corporation, China) according to the procedures listed in our previous works, too [25, 49]. A Q800 solids DMA analyzer (TA Corporation, USA) was employed to conduct the tests within a stretching mode (frequency: 1 Hz) and a temperature range from −50°C to 200°C (heating rate: 5°C min^−1^). An STA 409 PC thermogravimetry instrument from Netzsch Corporation (Germany) was used to perform TGA tests at a 20°C min^−1^ heating rate under *N*_2_. About 10 mg sample powders were used to performed the tests within a temperature range from 35 to 700°C. Tensile properties of samples were detected on an SANS7 CMT‐4304 universal instrument from Shenzhen Xinsansi Jiliang Instrument Corporation (China) with a speed of 5.0 mm min^−1^ and gauge length of 40 mm. For each sample, five specimens were tested at room temperature to calculate the average values. Molecular weights of samples were determined by GPC with a Waters Alliance system equipped with a refractive index detector (Waters Corporation, USA). The measurements were performed at 35°C using tetrahydrofuran as mobile phase at a flow rate of 1.0 mL min^−1^. The sample solutions were filtered through a PTFE syringe with a filter size of 0.45 µm before injection, and polystyrene standards were used for calibration. Antibacterial activity was evaluated according to the “Measurement of Antibacterial Activity on Plastics” standard (ISO 22196:2007, IDT). Recycling EPP_6_T_4_ films (Ø 40 mm) were inoculated with *Streptococcus mutans* and incubated at 37°C, and the colony‐forming units were quantified by the plate‐count method [50, 51]. Additionally, cell viability was assessed via CCK‐8 assay (C6005, NCM Biotech): L929 cells (10000 cells/well) were seeded in 96‐well plates, adhered overnight, and incubated with sample extracts (100%, 75%, 50%, 25%) for 24 h, then treated with CCK‐8 solution (37°C, 2 h dark incubation), finally absorbance at 450 nm (reference 650 nm) was measured to calculate viability relative to the control. Indirect contact cytotoxicity was determined by LDH assay (C0018, Beyotime Biotechnology): treated as the viability assay, supernatants (spontaneous LDH) and cell lysates (maximum LDH) were collected after 24 h, reacted with LDH working solution (25 min dark incubation at room temperature), and absorbance was measured to calculate cytotoxicity percentage. Shape memory properties were also tested by the DMA instrument. Typically, the sample was heated to 120°C and a constant stress of 0.4 MPa was applied to change its shape. Afterwards, the sample was cooled to 25°C at a rate of 5°C min^−1^ and the stress was removed, followed by heating the sample to 100°C with a rate of 5°C min^−1^ and keeping at this temperature for 40 min. For the life‐cycle assessment, the SimaPro software and Ecoinvent database were employed for modeling, and the model was quantified using the ReCiPe assessment method [8].

## Author Contributions

C.G. Liu. conceived the concept; C.G. Liu, H. Zhou, Y. Tan, J.F. Yao, and P. Zhao wrote and revised the paper; C.G. Liu, Y. Tan, H. Zhou, T.Y. Tang, and designed the experiments; H. Zhou, Y. Tan, C.W. Lu, Y.L. Li, T.Y. Tang, Z. Yang, Y. Li, X.H. Chen, Y.Y. Jiang, Z. C. Cai, and P. Zhao. conducted the experiments and analyzed experimental results.

## Conflicts of Interest

The authors declare no conflicts of interest.

## Supporting information




**Supporting File 1**: advs75006‐sup‐0001‐MovieS1.mp4.

**Supporting File 2**: advs75006‐sup‐0002‐MovieS2.mp4.

**Supporting File 3**: advs75006‐sup‐0003‐SuppMat.docx.

## Data Availability

The data that support the findings of this study are available from the corresponding author on request.
