# Supplementary material for: High‐Performance Recycling Biobased Photopolymers for 3D Printing
Source: Adv Sci (Weinh). 2026 Mar 25;13(32):e75006. doi: 10.1002/advs.75006 (PMC13252653; doi:10.1002/advs.75006)
Supplement: Supplementary file 3 — Supporting File 3: advs75006‐sup‐0003‐SuppMat.docx. [file ADVS-13-e75006-s003.docx]

Supporting Information

High-performance recycling biobased photopolymers for 3D printing

Hang Zhou^#^, Yi Tan^#^, Chuanwei Lu^#^, Yanlin Li, Tianyi Tang, Zheng Yang, Ying Li, Xuehan Chen, Jianfeng Yao^*^, Zhengchun Cai, Yuanyuan Jiang, Ping Zhao^*^, Chengguo Liu^*^

#These Authors contributed equally to this work.

*Corresponding authors: Jianfeng Yao, jfyao@njfu.edu.cn; Ping Zhao, hypzhao2022@163.com; Chengguo Liu, liuchengguo@njfu.edu.cn

**This PDF file includes:**

Supplementary Tables S1 to S15

Supplementary Figures S1 to S20

**Table of Contents**

[S1. Synthetic route of eugenol-based prepolymers and resin compositions 3](#_Toc223619266)

[S2. Optimalization of experimental conditions 4](#_Toc223619267)

[S3. Structural characterization 5](#_Toc223619268)

[S4. Physical properties and curing kinetic parameters 10](#_Toc223619269)

[S5. Photopolymerization kinetics curves of EDPT and EPPT 11](#_Toc223619270)

[S6. Thermal and mechanical properties of EDPT and EPPT resins 12](#_Toc223619271)

[S7. Stress relaxation behaviors of EDPT and EPPT 14](#_Toc223619272)

[S8. Creep resistance performance of the EPP_6_T_4_ 16](#_Toc223619273)

[S9. Comparison of recycling efficiency of material mass in different open-loop recycling systems 17](#_Toc223619274)

[S10. Molecular weights, curing kinetic parameters, and mechanical/thermal properties of the pristine and recycled resins 17](#_Toc223619275)

[S11. Comparison of 3D printing performance between EPP_6_T_4_ and petroleum-based commercial resin 18](#_Toc223619276)

[S12. Cytotoxicity tests for EPP_6_T_4_ 19](#_Toc223619277)

[S13. Parameters in the shape memory cycle tests 20](#_Toc223619278)

[S14. Process of life-cycle assessment (LCA) 21](#_Toc223619279)

S1. Synthetic route of eugenol-based prepolymers and resin compositions





**Figure S1** Synthetic route of EPP and EDP.

**Table S1** Compositions of EDPT and EPPT resins

| **Sample** | **EDP**  **(g)** | **EPP**  **(g)** | **THFMA***  **(g)** | **Darocur 1173**  **(g)** |
| --- | --- | --- | --- | --- |
| EDP_8_T_2_ | 16.0 | / | 4.0 | 0.4 |
| EDP_7_T_3_ | 14.0 | / | 6.0 | 0.4 |
| EDP_6_T_4_ | 12.0 | / | 8.0 | 0.4 |
| EPP_8_T_2_ | / | 16.0 | 4.0 | 0.4 |
| EPP_7_T_3_ | / | 14.0 | 6.0 | 0.4 |
| EPP_6_T_4_ | / | 12.0 | 8.0 | 0.4 |

*THFMA: tetrahydrofurfuryl methacrylate.

S2. Optimalization of experimental conditions

**Figure S2** FT-IR spectra of isocyanate group during the synthesis of EPP.

**Figure S3** Stress–strain curves of EPP_6_T_4_ with varying ratios of EP to ICEMA.

S3. Structural characterization

**S3.1 FT-IR characterization**

FT-IR spectra of eugenol, eugenol-based phenols (ED and EP), and eugenol-based prepolymers (EDP and EPP) are depicted in Figure S4–S5. In the spectra of eugenol and EP, the absorption peak at 3447 cm⁻^1^ was attributed to the stretching vibration of hydroxyl (–OH) group. However, this peak disappeared in the spectrum of EPP, indicating the successful reaction of –OH during the synthesis process. The absorption band at 3360 cm⁻^1^ corresponded to the N–H stretching vibration, confirming the formation of carbamate structure in EPP. The peaks at 2978 and 2950 cm⁻^1^ were ascribed to the C–H stretching vibrations of methyl and methylene groups. The peak at 1718 cm⁻^1^ in the EP spectrum was assigned to the stretching vibration of carbonyl (C=O) group from PEMTP. However, this peak shifted to 1733 cm^-^^1^ and its intensity became much larger, indicating the formation of new carbamate, too. Additionally, the peak at 1640 cm⁻^1^ in EPP corresponded to the stretching vibration of C=C in acrylate, as there was no such peak in EP. The spectra of ED and EDP showed similar results to those of EP and EPP.

**Figure S4** FT-IR spectra of eugenol, EP, and EPP.

**Figure S5** FT-IR spectra of eugenol, ED, and EDP.

**S3.2 ^1^H NMR characterization**

^1^H NMR spectra of eugenol, eugenol-based phenols, and eugenol-based prepolymers are shown in Figure S6–S10. As for eugenol, the signals at 6.5–7.0 ppm corresponded to aromatic protons, which appeared as multiplets due to the influence of substituents and vicinal coupling with adjacent protons.^[1]^ The broad and irregular signals within 5.0–6.0 ppm were attributed to the protons of hydroxyl (–OH) and allyl C=C group.^[2]^ The signals observed at 3.5–4.0 ppm were assigned to the protons of methoxy (–OCH₃) group, which generally appeared as a sharp peak due to the absence of neighboring protons.^[3]^ In the spectrum of EP, the aromatic proton ensemble appeared at 6.5–7.0 ppm, while the peaks at 5.77 and 3.83 ppm were assigned to the labile hydroxyl and methoxy (−OCH_3_) protons, respectively.^[2]^ These resonances were slightly shifted relative to native eugenol, although their multiplicity was preserved. Three peaks were observed between 2.8 and 2.4 ppm (each was 2H after integrating), which can be attributed to the methylene groups of EP.^[4]^ In the ^1^H NMR spectrum of EPP, the hydroxyl proton signal was absent, indicating its consumption during the functionalization. Most resonances corresponding to EP framework (H-1 through H-7) retained similar chemical shifts and multiplicities, confirming preservation of the core skeleton; several new signals, however, appeared and were diagnostic of the expected derivatization.^[5]^ A triplet peak at 5.83 ppm (integral = 1H) was assigned to an amide (–NH) proton, whose multiplicity reflects coupling with adjacent methylene protons. Two vinyl methylene resonances at 6.15 and 5.59 ppm (each 1H) corresponded to terminal C=C–CH_2_ protons.^[6]^ Signals at 4.26 ppm (2H, t) and 3.54 ppm (2H, q) were assigned to oxygen- and nitrogen-linked methylene protons, respectively, and a singlet at 1.95 ppm (3H) corresponded to the methyl group from methacrylate.^[7]^ The spectra of ED and EDP also demonstrated similar results to those of EP and EPP.

**Figure S6** ^1^H NMR spectrum of eugenol.

**Figure S7** ^1^H NMR spectrum of EP.

**Figure S8** ^1^H NMR spectrum of EPP.

**Figure S9** ^1^H NMR spectrum of ED.

**Figure S10** ^1^H NMR spectrum of EDP.

S4. Physical properties and curing kinetic parameters

**Table S2** Physical properties and curing kinetic parameters of EDPT and EPPT.

| **Sample** | ***V*_s_ ^a^**  **(MPa∙s)** | ***C*_gel_ ^b^**  **(%)** | ***C*_bio_ ^c^**  **(%)** | ***C*_DCB_ ^d^**  **(mol g^−1^)** | **Δ*V* ^e^**  **(%)** | ***α*_f_ ^f^**  **(%)** | ***R*_p_ ^g^**  **(s^-1^)** |
| --- | --- | --- | --- | --- | --- | --- | --- |
| EDP_8_T_2_ | 481±4 | 95.3±0.2 | 43.9 | 0.0313 | 9.01±0.15 | 63.5 | 0.114 |
| EDP_7_T_3_ | 247±3 | 96.5±0.3 | 45.8 | 0.0274 | 8.91±0.30 | 71.8 | 0.102 |
| EDP_6_T_4_ | 115±1 | 97.9±0.1 | 47.8 | 0.0235 | 8.94±0.47 | 82.3 | 0.182 |
| EPP_8_T_2_ | 580±1 | 97.6±0.2 | 41.6 | 0.0279 | 15.9±0.2 | 65.3 | 0.0780 |
| EPP_7_T_3_ | 348±1 | 98.1±0.2 | 43.8 | 0.0244 | 10.5±0.3 | 71.2 | 0.107 |
| EPP_6_T_4_ | 103±2 | 98.5±0.1 | 46.1 | 0.0210 | 8.83±0.13 | 75.7 | 0.130 |

*^a^* Viscosity at 25 ℃. *^b^* Gel content. *^c^* Biobased content. *^d^* Content of dynamic covalent bond. *^e^* Volumetric shrinkage. *^f^* Final C=C conversion. *^g^* Maximum C=C conversion rate.

S5. Photopolymerization kinetics curves of EDPT and EPPT

**Figure S11** (a, b) C=C conversions and (c, d) their rates of EDPT and EPPT.

S6. Thermal and mechanical properties of EDPT and EPPT resins

**Table S3** Thermal properties of EDPT and EPPT resins.

| **Sample** | ***C*_HS_ ^a^**  **(%)** | ***E’*_25_ ^b^**  **(GPa)** | ***T*_g_ ^c^**  **(****°C)** | ***E’*_Tg+40_ ^d^**  **(MPa)** | ***ν*_e_ ^e^**  **(10^3^mol m^−3^)** | ***T*_5_ ^f^**  **(°C )** | ***T*_max1_ ^g^**  **(°C )** | ***T*_max2_ ^h^**  **(°C )** | ***w*_char_ ^i^**  **(%)** |
| --- | --- | --- | --- | --- | --- | --- | --- | --- | --- |
| EDP_8_T_2_ | 83.4 | 2.05 | 73.6 | 14.8 | 1.71 | 237 | 276 | 394 | 8.73 |
| EDP_7_T_3_ | 85.4 | 2.16 | 75.4 | 12.6 | 1.45 | 240 | 274 | 392 | 4.21 |
| EDP_6_T_4_ | 87.5 | 2.21 | 75.4 | 9.90 | 1.14 | 245 | 275 | 392 | 5.64 |
| EPP_8_T_2_ | 77.8 | 2.50 | 76.7 | 19.8 | 2.27 | 193 | 292 | 430 | 9.98 |
| EPP_7_T_3_ | 80.6 | 2.17 | 79.0 | 15.3 | 1.74 | 207 | 290 | 430 | 8.67 |
| EPP_6_T_4_ | 83.4 | 2.40 | 83.2 | 12.5 | 1.40 | 220 | 290 | 429 | 7.36 |

*^a^* Hard segment content. *^b^* Storage modulus at 25°C. *^c^* Glass transition temperature. *^d^* Storage modulus at *T*_g_+ 40°C. *^e^* Cross-link density. *^f^* 5% weight loss temperature. *^g^* Maximum thermal decomposition temperatures. *^h^* Maximum thermal decomposition temperatures. *^i^* Char yield.

**Table S4** Mechanical properties of EDPT and EPPT resins.

| **Sample** | ***σ* ^a^**  **(MPa)** | ***E* ^b^**  **(GPa)** | ***ε* ^c^**  **(%)** |
| --- | --- | --- | --- |
| EDP_8_T_2_ | 40.3±1.5 | 0.784±0.015 | 9.91±0.91 |
| EDP_7_T_3_ | 44.5±2.4 | 0.822±0.011 | 10.9±0.6 |
| EDP_6_T_4_ | 51.6±2.2 | 0.894±0.007 | 12.6±1.1 |
| EPP_8_T_2_ | 54.7±2.1 | 0.941±0.008 | 8.32±0.79 |
| EPP_7_T_3_ | 59.0±2.3 | 1.02±0.01 | 8.02±0.61 |
| EPP_6_T_4_ | 68.0±2.7 | 1.41±0.01 | 7.85±0.81 |

*^a^* Tensile strength. *^b^* Tensile modulus. *^c^* Elongation at the break.

**Figure S12** Typical stress–strain curve of a petroleum-based commercial resin.

**Figure S13** Storage modulus and loss factor of a petroleum-based commercial resin.

**Table S5** Mechanical and thermal properties of EPP_6_T_4_ and petroleum-based commercial resin.

| **Sample** | ***σ* ^a^**  **(MPa)** | ***E* ^b^**  **(GPa)** | ***ε* ^c^**  **(%)** | ***T*_g_ ^d^**  **(°C)** |
| --- | --- | --- | --- | --- |
| EPP_6_T_4_ | 68.0 | 1.41 | 7.85 | 83.2 |
| Petroleum-based commercial resin | 56.7 | 1.27 | 6.67 | 87.1 |

*^a^* Tensile strength. *^b^* Tensile modulus. *^c^* Elongation at the break. *^d^* Glass transition temperature.

S7. Stress relaxation behaviors of EDPT and EPPT

Activation energy (*E*_a_) of the cured samples was calculated based on the Arrhenius equation:

$\ln\tau=\frac{E_{a}}{RT}+ln \tau₀$ (5)

where *τ*₀ is the pre-exponential factor corresponding to the characteristic relaxation time at infinite temperature, *E*_a_ is the activation energy, *R* is the universal gas constant, and *T* is the absolute temperature.^[8]^

**Table S6** Relaxation times of EDPT and EPPT.

| **Sample** | ***R*_t1_ ^a^**  **(min)** | ***R*_t2_ ^b^**  **(min)** |
| --- | --- | --- |
| EDP_8_T_2_ | 21.9 | / |
| EDP_7_T_3_ | 19.4 | / |
| EDP_6_T_4_ | 15.5 | / |
| EPP_8_T_2_ | / | 4.31 |
| EPP_7_T_3_ | / | 4.01 |
| EPP_6_T_4_ | / | 3.71 |

*^a^* Relaxation time at 180 ^o^C. *^b^* Relaxation time at 200 ^o^C.

**Figure S14** Stress relaxation curves of (a) EDP_6_T_4_ and (b) EPP_6_T_4_ at different temperatures.

**Table S7** Relaxation times (*R*_t_) of EDP_6_T_4_ and EPP_6_T_4_ at different temperatures.

| **Sample** | ***R*_t_ (190 °C)**  **(min)** | ***R*_t_ (200 °C)**  **(min)** | ***R*_t_ (210 °C)**  **(min)** | ***R*_t_ (220 °C)**  **(min)** |
| --- | --- | --- | --- | --- |
| EDP_6_T_4_ | 5.55 | 3.33 | 2.04 | 1.49 |
| EPP_6_T_4_ | 14.2 | 4.58 | 3.45 | 2.62 |

S8. Creep resistance performance of the EPP_6_T_4_

**Figure S15** Creep resistance performance of EPP_6_T_4_ at 40–60 °C.

 **Figure S16** Creep resistance performance of EPP_6_T_4_ at 80–100 °C.

S9. Comparison of recycling efficiency of material mass in different open-loop recycling systems

**Table S8** Recycling efficiency of material mass in different open-loop recycling systems.

| **Entry** | **Recycling efficiency of material mass**  **(%)** | **Ref.** |
| --- | --- | --- |
| 1 | 5–17 | [9] |
| 2 | 15 | [10] |
| 3 | 7.4–16.7 | [11] |
| 4 | 22.9–27.5 | This work |

S10. Molecular weights, curing kinetic parameters, and mechanical/thermal properties of the pristine and recycled resins

**Table S9** Molecular weights, curing kinetic parameters, and mechanical properties of the pristine and recycled EPP_6_T_4_.

| **Sample** | ***M*_w_ ^a^**  **(g/mol)** | ***M*_n_ ^b^**  **(g/mol)** | ***PDI* ^c^** | ***α*_f_ ^d^**  **(%)** | ***R*_p_ ^e^**  **(s^-1^)** | ***σ ^f^***  **(MPa)** | ***E ^g^***  **(GPa)** | ***ε* ^h^**  **(%)** |
| --- | --- | --- | --- | --- | --- | --- | --- | --- |
| Pristine | 1150 | 532 | 2.16 | 75.7 | 0.130 | 72.5±1.2 | 1.43±0.02 | 7.93±0.28 |
| Recycle-1^st^ | 1123 | 528 | 2.12 | 83.8 | 0.118 | 71.7±0.5 | 1.41±0.01 | 7.59±0.65 |
| Recycle-2^nd^ | 1190 | 584 | 2.03 | 79.4 | 0.128 | 68.7±1.8 | 1.38±0.01 | 8.10±0.36 |
| Recycle-3^rd^ | 1228 | 670 | 1.83 | 77.5 | 0.083 | 67.4±1.6 | 1.38±0.02 | 7.15±0.41 |

*^a^* Weight-average molecular weight*. ^b^* Number-average molecular weight*. ^c^* Polydispersity. *^d^* Final C=C conversion. *^e^* Maximum C=C conversion rate. *^f^* Tensile strength. *^g^* Tensile modulus. *^h^* Elongation at break.

**Table S10** Thermal properties of the pristine and recycled EPP_6_T_4_.

| **Sample** | ***E’*_25_ ^a^**  **(GPa)** | ***T*_g_ ^b^**  **(°C)** | ***E’*_Tg+40_ ^c^**  **(MPa)** | ***ν*_e_ ^d^**  **(10^3^mol/m^3^)** | ***T*_5_ ^e^**  **(°C)** | ***T*_max1_ ^f^**  **(°C)** | ***T*_max2_ ^g^**  **(°C)** | ***w*_char_ ^h^**  **(%)** |
| --- | --- | --- | --- | --- | --- | --- | --- | --- |
| Pristine | 2.40 | 83.2 | 12.5 | 1.40 | 226 | 281 | 425 | 6.66 |
| Recycle-1^st^ | 2.53 | 86.7 | 14.2 | 1.48 | 244 | 281 | 425 | 6.03 |
| Recycle-2^nd^ | 2.62 | 87.5 | 14.9 | 1.50 | 237 | 278 | 426 | 5.35 |
| Recycle-3^rd^ | 2.81 | 88.7 | 15.5 | 1.56 | 232 | 277 | 423 | 3.48 |

*^a^* Storage modulus at 25°C. *^b^* Glass transition temperature. *^c^* Storage modulus at *T*_g_+ 40°C. *^d^* Cross-link density. *^e^* 5% weight loss temperature. *^f^* Maximum thermal decomposition temperatures. *^g^* Maximum thermal decomposition temperatures. *^h^* Char yield.

S11. Comparison of 3D printing performance between EPP_6_T_4_ and petroleum-based commercial resin

**Table S11** Comparison of mechanical properties between 3D-printed EPP_6_T_4_ tensile specimens and commercial resin.

| **Sample** | **EPP_6_T_4_** | | **Petroleum-based commercial resin** | |
| --- | --- | --- | --- | --- |
|  | **XY** | **Z** | **XY** | **Z** |
| Tensile stress (MPa) | 55.3 | 56.1 | 39.3 | 29.7 |
| Tensile modulus (GPa) | 2.14 | 2.10 | 1.07 | 1.15 |
| Elongation at break (%) | 6.71 | 4.86 | 10.4 | 14.5 |


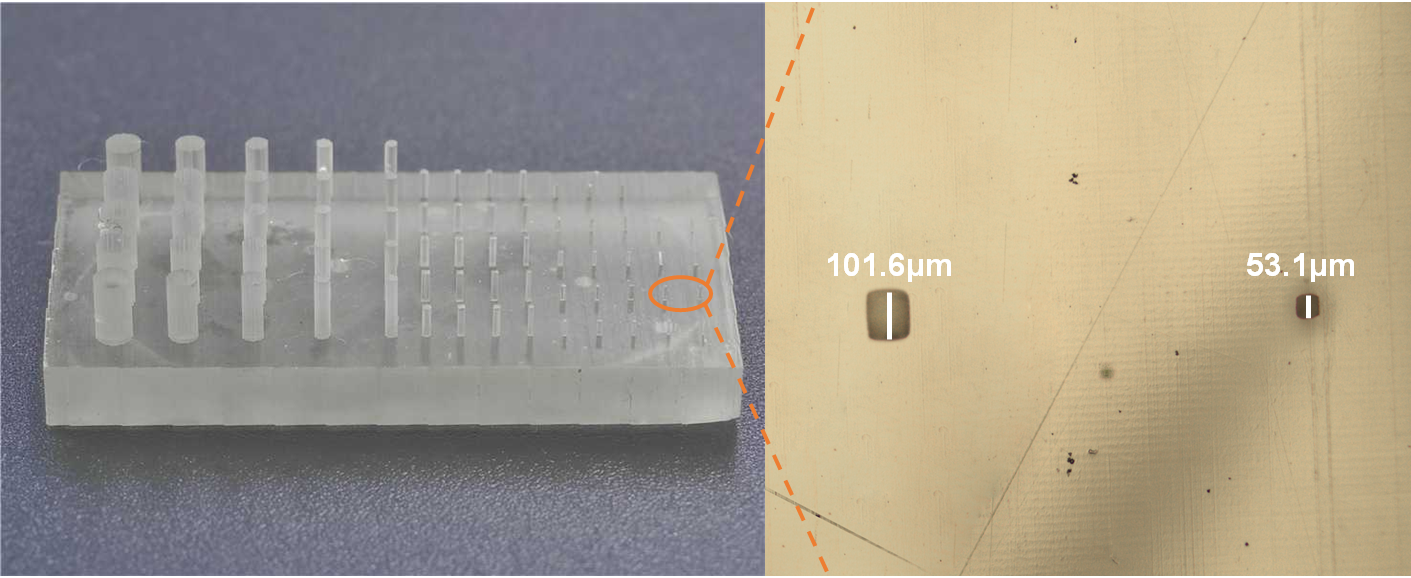


**Figure S17.** XY-axis precision demonstration of EPP_6_T_4_ for 3D printed physical objects.

S12. Cytotoxicity tests for EPP_6_T_4_


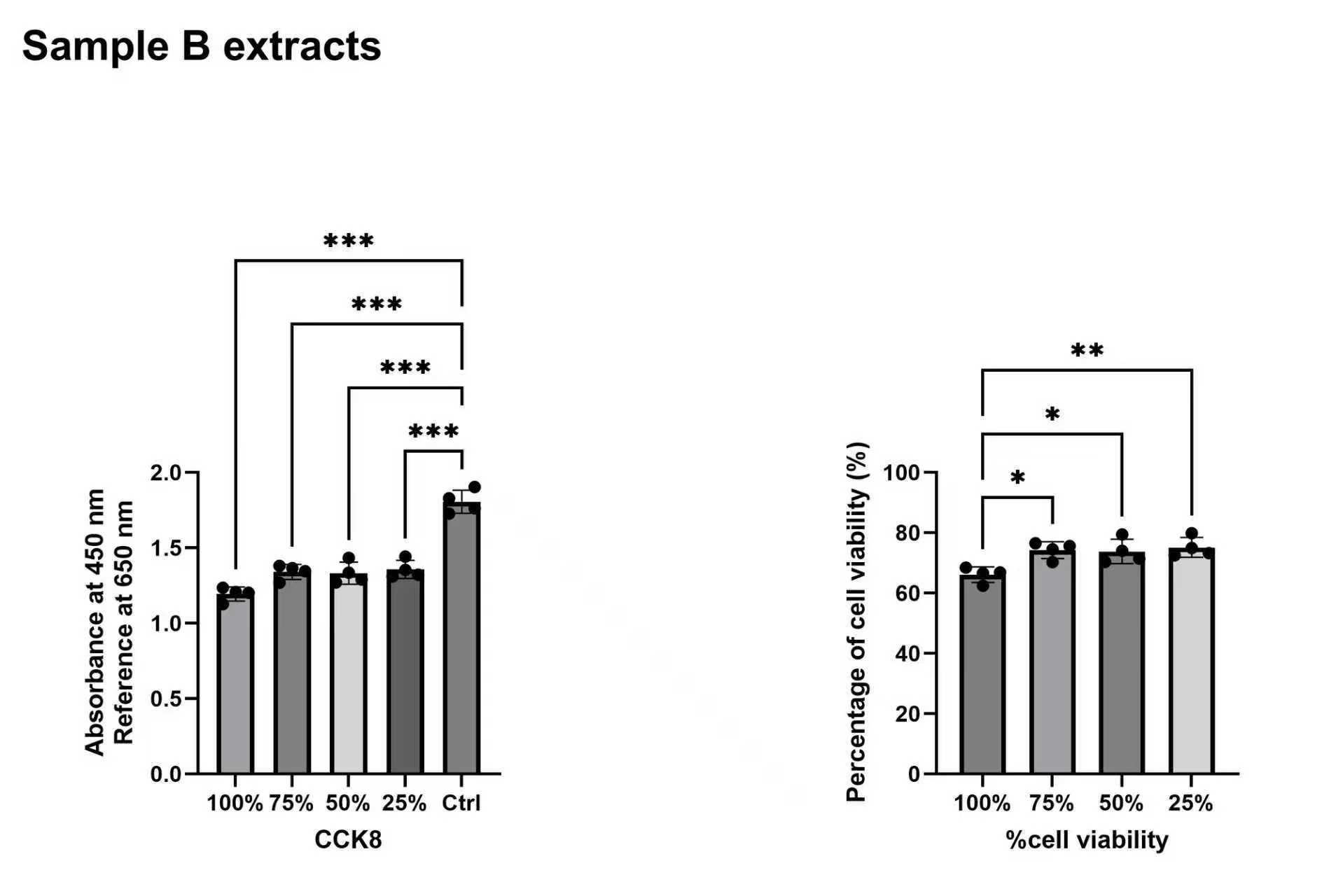


**Figure S18.** Indirect contact cell viability determined by CCK‑8 assay.


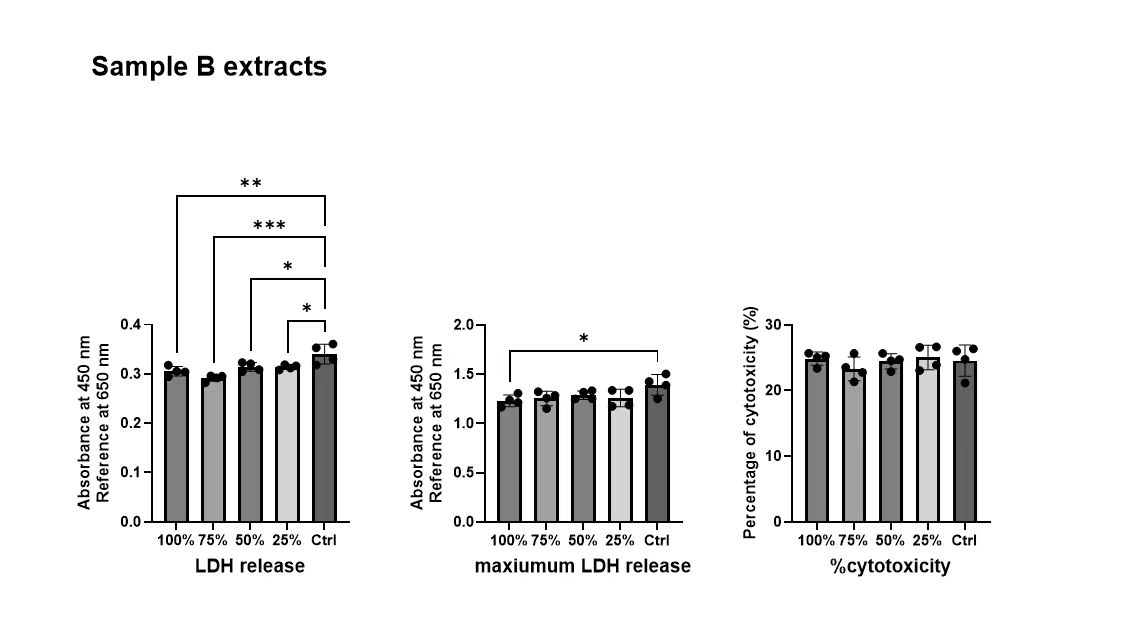


**Figure S19.** Indirect contact cytotoxicity determined by LDH assay.

S13. Parameters in the shape memory cycle tests

**Table S12** Parameters in the shape memory cycle tests.

|  | ***ε*_unload_ ^a^**  **(%)** | ***ε*_load_ ^b^**  **(%)** | ***ε*_rec_ ^c^**  **(%)** | ***R*_f_ ^d^**  **(%)** | ***R*_r_ ^e^**  **(%)** |
| --- | --- | --- | --- | --- | --- |
| 1^st^ cycle | 4.56 | 5.61 | 1.89 | 83.2 | 65.0 |
| 2^nd^ cycle | 4.63 | 5.70 | 1.93 | 84.6 | 64.9 |
| 3^rd^ cycle | 4.70 | 5.76 | 2.01 | 83.3 | 59.5 |
| 4^th^ cycle | 4.81 | 5.84 | 2.07 | 84.1 | 63.9 |

*^a^* Fixed strain. *^b^* Initial strain. *^c^* Recovery strain. *^d^* Shape fixity ratio. *^e^* Shape recovery ratio.

S14. Process of life-cycle assessment (LCA)


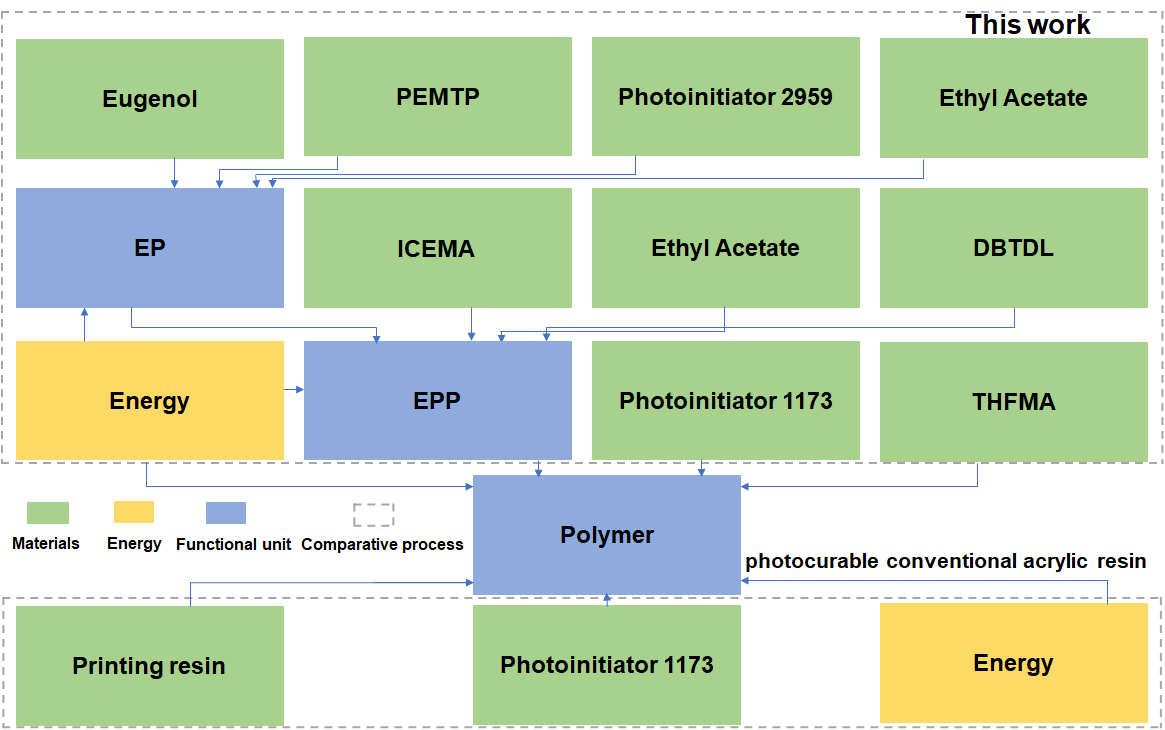


**Figure S20.** System boundary diagram of material and energy flows for this work and the photocurable conventional acrylic resin in the life-cycle assessment (LCA).^[12]^

**Table S13** Life cycle inventory of this work.

| **Stage** | **Status** | **Name** | **Value** | **Unit** | **Category** |
| --- | --- | --- | --- | --- | --- |
| Preparation of EP | Input | Eugenol | 1.00 | kg | Materials |
|  |  | PEMTP | 0.743 | kg | Materials |
|  |  | Photoinitiator 2959 | 0.0174 | kg | Materials |
|  |  | Ethyl Acetate | 1.82 | kg | Materials |
|  |  | Ultraviolet Light | 25.9 | kJ | Energy |
|  |  | Heat Energy from Oil Bath | 165 | kJ | Energy |
|  | Output | consumed solvent Ethyl Acetate | 0.18 | kg | Waste |
|  |  | EP | 1.60 | kg | Intermediate product |
|  |  | Ethyl Acetate | 1.64 | kg | Recycling |
| Preparation of EPP | Input | EP | 1.60 | kg | Materials |
|  |  | ICEMA | 0.812 | kg | Materials |
|  |  | Ethyl Acetate | 1.80 | kg | Materials |
|  |  | DBTDL | 0.0231 | kg | Materials |
|  |  | Heat Energy from Oil Bath | 665 | kJ | Energy |
|  | Output | consumed solvent Ethyl Acetate | 0.24 | kg | Waste |
|  |  | EPP | 2.30 | kg | Intermediate product |
|  |  | Ethyl Acetate | 1.56 | kg | Recycling |
| First printing process | Input | EPP | 2.30 | kg | Materials |
|  |  | THFMA | 1.53 | kg | Materials |
|  |  | Photoinitiator 1173 | 0.0771 | kg | Materials |
|  |  | Ultraviolet Light | 900 | kJ | Energy |
|  |  | Heat energy from oven | 1500 | kJ | Energy |
|  | Output | EPP_6_T_4_ | 3.90 | kg | Intermediate product |
| Recycling process  (repeat three times) | Input | EPP_6_T_4_ | 3.90 | kg | Materials |
|  |  | ICEMA | 3.23 | kg | Materials |
|  |  | EP | 6.15 | kg | Materials |
|  |  | Heat Energy from Oil Bath | 1210 | kJ | Energy |
|  |  | THFMA | 5.13 | kg | Materials |
|  |  | Photoinitiator 1173 | 0.0912 | kg | Materials |
|  | Output | Recycled Photocurable Liquid Resin | 18.3 | kg | Intermediate product |
| Printing of recycled resin  (repeat three times) | Input | Recycled Photocurable Liquid Resin | 18.3 | kg | Materials |
|  |  | Ultraviolet Light | 4200 | kJ | Energy |
|  | Output | Resin after three recycling cycles | 18.3 | kg | Intermediate product |

**Table S14** Detailed comparative characterization results.

| **Impact categories** | **Abbreviation** | **This work** | **photocurable conventional acrylic resin[12]** | **Unit** |
| --- | --- | --- | --- | --- |
| Global warming potential | GWP | 3.61 | 7.58 | kg CO_2_ eq |
| Ozone depletion | OD | 1.41E-06 | 1.58E-06 | kg CFC_11_ eq |
| Ionizing radiation | IR | 0.152 | 0.310 | kBq Co-60 eq |
| Particle matter formation | PMF | 0.00629 | 0.00917 | kg PM_2.5_ eq |
| Terrestrial acidification | TA | 0.0148 | 0.0199 | kg SO_2_ eq |
| Freshwater eutrophication potential | FEP | 0.00140 | 0.0256 | kg P eq |
| Marine eutrophication potential | MEP | 0.000212 | 0.000360 | kg N eq |
| Terrestrial ecotoxicity | TE | 48.4 | 190 | kg 1,4-DB eq |
| Freshwater ecotoxicity | FE | 0.163 | 0.317 | kg 1,4-DB eq |
| Marine ecotoxicity | ME | 0.253 | 0.575 | kg 1,4-DB eq |
| Human carcinogenic toxicity | HCT | 0.491 | 1.21 | kg 1,4-DB eq |
| Human noncarcinogenic toxicity | HNCT | 4.06 | 6.70 | kg 1,4-DB eq |
| Mineral resource scarcity | MRS | 0.0208 | 0.0229 | kg Cu eq |
| Fossil resource scarcity | FRS | 1.19 | 2.86 | kg Oil eq |
| Water consumption | WC | 0.0472 | 0.0401 | m^3^ |

**Table S15** Summarized damages for human health, ecosystems, and resources.

| **Damage**  **categories** | **Weighted normalization in LCA (unit: Pt)** | |
| --- | --- | --- |
|  | **This work** | **photocurable conventional acrylic resin** |
| Human health | 0.0133 | 0.0154 |
| Ecosystems | 0.0145 | 0.0167 |
| Resources | 2.54 | 2.86 |
| Total | 2.57 | 2.89 |

**References**

[1] R. J. Abraham, L. Griffiths, M. Perez, *Magn. Reson. Chem.* **2014**, *52* (7), 395.

[2] P. Charisiadis, V. G. Kontogianni, C. G. Tsiafoulis, A. G. Tzakos, M. Siskos, I. P. Gerothanassis, *Molecules* **2014**, *19* (9), 13643.

[3] L. A. De Souza, W. M. Tavares, A. P. M. Lopes, M. M. Soeiro, W. B. De Almeida, *Chem. Phys. Lett.* **2017**, *676*, 46.

[4] M. DeCamp, L. DeFlores, J. McCracken, A. Tokmakoff, K. Kwac, M. Cho, *J. Phys. Chem. B* **2005**, *109* (21), 11016.

[5] R. Ding, Y. Du, R. B. Goncalves, L. F. Francis, T. M. Reineke, *Polym. Chem.* **2019**, *10* (9), 1067.

[6] M. Maturi, C. Spanu, E. Maccaferri, E. Locatelli, T. Benelli, L. Mazzocchetti, L. Sambri, L. Giorgini, M. C. Franchini, *ACS Sustain. Chem. Eng.* **2023**, *11* (49), 17285.

[7] G. Gamov, V. Aleksandriiskii, V. Sharnin, *J. Mol. Liq.* **2017**, *231*, 238.

[8] H. Zhou, C. G. Liu, J. Huang, Y. L. Li, G. Q. Zhu, C. W. Lu, J. F. Yao, H. J. Xu, P. Zhao, *Ind. Crops Prod.* **2025**, *224*, 120299.

[9] G. D. Zhu, Y. Hou, J. Xu, N. Zhao, *Adv. Funct. Mater.* **2021**, *31* (9), 2007173.

[10] Z. Q. Chen, M. Yang, M. K. Ji, X. Kuang, H. J. Qi, T. J. Wang, *Mater. Des.* **2021**, *197*, 109189.

[11] G. Q. Zhu, J. S. Zhang, J. Huang, Y. H. Qiu, M. T. Liu, J. N. Yu, C. G. Liu, Q. Q. Shang, Y. Hu, L. H. Hu, *Chem. Eng. J.* **2023**, *452*, 139401.

[12] R. Rajan, E. Rainosalo, M. Lebedevaite, J. Ostrauskaite, V. Talacka, *Master thesis*, Centria University of Applied Sciences **2022**.
